# Finding biomarkers in non-model species: literature mining of transcription factors involved in bovine embryo development

**DOI:** 10.1186/1756-0381-5-12

**Published:** 2012-08-29

**Authors:** Nicolas Turenne, Evgeniy Tiys, Vladimir Ivanisenko, Nikolay Yudin, Elena Ignatieva, Damien Valour, Séverine A Degrelle, Isabelle Hue

**Affiliations:** 1 INRA, SenS, UR1326, IFRIS, Champs-sur-Marne, F-77420, France; 2 Sector of Computational Proteomics, Institute of Cytology and Genetics, 10 Lavrentyev Ave, Novosibirsk, 630090, Russia; 3 INRA, UMR1198 Biologie du Développement et Reproduction, Jouy-en-Josas, F-78352, France; 4 ENVA, Maisons Alfort, F-94704, France; 5 Laboratory of Animal Molecular Genetics, Institute of Cytology and Genetics, 10 Lavrentyev Ave, Novosibirsk, 630090, Russia; 6 Laboratory of Evolutionary Bioinformatics and Theoretical, Institute of Cytology and Genetics, 10 Lavrentyev Ave, Novosibirsk, 630090, Russia

## Abstract

**Background:**

Since processes in well-known model organisms have specific features different from those in *Bos taurus*, the organism under study, a good way to describe gene regulation in ruminant embryos would be a species-specific consideration of closely related species to cattle, sheep and pig. However, as highlighted by a recent report, gene dictionaries in pig are smaller than in cattle, bringing a risk to reduce the gene resources to be mined (and so for sheep dictionaries). Bioinformatics approaches that allow an integration of available information on gene function in model organisms, taking into account their specificity, are thus needed. Besides these closely related and biologically relevant species, there is indeed much more knowledge of (i) trophoblast proliferation and differentiation or (ii) embryogenesis in human and mouse species, which provides opportunities for reconstructing proliferation and/or differentiation processes in other mammalian embryos, including ruminants. The necessary knowledge can be obtained partly from (i) stem cell or cancer research to supply useful information on molecular agents or molecular interactions at work in cell proliferation and (ii) mouse embryogenesis to supply useful information on embryo differentiation. However, the total number of publications for all these topics and species is great and their manual processing would be tedious and time consuming. This is why we used text mining for automated text analysis and automated knowledge extraction. To evaluate the quality of this “mining”, we took advantage of studies that reported gene expression profiles during the elongation of bovine embryos and defined a list of transcription factors (or TF, n = 64) that we used as biological “gold standard”. When successful, the “mining” approach would identify them all, as well as novel ones.

**Methods:**

To gain knowledge on molecular-genetic regulations in a non model organism, we offer an approach based on literature-mining and score arrangement of data from model organisms. This approach was applied to identify novel transcription factors during bovine blastocyst elongation, a process that is not observed in rodents and primates. As a result, searching through human and mouse corpuses, we identified numerous bovine homologs, among which 11 to 14% of transcription factors including the gold standard TF as well as novel TF potentially important to gene regulation in ruminant embryo development. The scripts of the workflow are written in Perl and available on demand. They require data input coming from all various databases for any kind of biological issue once the data has been prepared according to keywords for the studied topic and species; we can provide data sample to illustrate the use and functionality of the workflow.

**Results:**

To do so, we created a workflow that allowed the pipeline processing of literature data and biological data, extracted from Web of Science (WoS) or PubMed but also from Gene Expression Omnibus (GEO), Gene Ontology (GO), Uniprot, HomoloGene, TcoF-DB and TFe (TF encyclopedia). First, the human and mouse homologs of the bovine proteins were selected, filtered by text corpora and arranged by score functions. The score functions were based on the gene name frequencies in corpora. Then, transcription factors were identified using TcoF-DB and double-checked using TFe to characterise TF groups and families. Thus, among a search space of 18,670 bovine homologs, 489 were identified as transcription factors. Among them, 243 were absent from the high-throughput data available at the time of the study. They thus stand so far for putative TF acting during bovine embryo elongation, but might be retrieved from a recent RNA sequencing dataset (Mamo et al. , 2012). Beyond the 246 TF that appeared expressed in bovine elongating tissues, we restricted our interpretation to those occurring within a list of 50 top-ranked genes. Among the transcription factors identified therein, half belonged to the gold standard (ASCL2, c-FOS, ETS2, GATA3, HAND1) and half did not (ESR1, HES1, ID2, NANOG, PHB2, TP53, STAT3).

**Conclusions:**

A workflow providing search for transcription factors acting in bovine elongation was developed. The model assumed that proteins sharing the same protein domains in closely related species had the same protein functionalities, even if they were differently regulated among species or involved in somewhat different pathways. Under this assumption, we merged the information on different mammalian species from different databases (literature and biology) and proposed 489 TF as potential participants of embryo proliferation and differentiation, with (i) a recall of 95% with regard to a biological gold standard defined in 2011 and (ii) an extension of more than 3 times the gold standard of TF detected so far in elongating tissues. The working capacity of the workflow was supported by the manual expertise of the biologists on the results. The workflow can serve as a new kind of bioinformatics tool to work on fused data sources and can thus be useful in studies of a wide range of biological processes.

## Background

### Mining context

Molecular-genetic data obtained from model organisms are widely used in studies of biological processes. The problem is that the processes studied in the well-known model organisms often have specific features different from those in related non-model organisms. For this reason, a single model organism cannot give a complete idea of a process of interest. Development of bioinformatics approaches that allow integration of available information on gene function in model organisms taking into account their specificity is therefore an important task. This task is relevant to almost all areas of biology, including biomedicine, veterinary, and agriculture. For example, studies of embryo development in ruminants are important to i) understand embryonic loss, especially in high producing dairy cows and ii) compare developmental processes across species. However, molecular-genetic data on ruminant embryo development are scant and blastocyst elongation is not observed in rodents and primates; as a result, the number of well-studied organisms to refer to is limited. A good way to describe gene regulation in embryo development from ruminants would be a species-specific consideration of closely related species to cattle, such as sheep or pig, along with the well-studied model organisms, such as human and mouse. The current study offers an approach for knowledge acquisition on molecular-genetic regulation in any organism based on literature mining and integration of data sources from model organisms. This approach was applied to identify transcription factors acting in ruminant embryo development. We analyzed data on trophoblast proliferation and differentiation, embryogenesis, stem cells and cancer concerning cattle, human, mouse, rat, sheep, pig and horse, that were reported in PubMed and WoS. As a result, we identified novel genes potentially important to gene regulation in ruminant embryo development. These results will be helpful to design further biological experiments on ruminant embryo development but a similar approach would be useful to study other biological processes using data sources from other model organisms in either animals or plants. Approaches to search for articles that describe molecular-genetic mechanisms underlying complex biological processes have been developed. For example, a service enabling literature search and ranking based on their biological relevance to gene sets has been presented recently [1]. However, our approach is better in tune with ongoing issues in text-mining such as feature selection and fusion of literature and biological databases [2]. In animal sciences, text-mining just starts to be in the scope of *in silico* methods [3] and TF Encyclopedia proves the interest around transcription factors in life sciences [4].

### Biological issue

**Figure 1:**
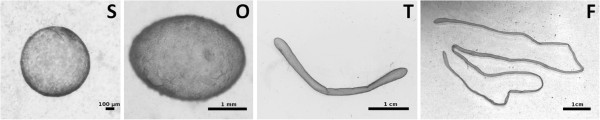
**Microscopic and macroscopic views of the bovine embryo.** Microscopic and macroscopic views of the bovine embryo during the elongation phase: spherical (**S**), ovoid (**O**), tubular (**T**) and filamentous (**F**) shapes appear sequentially from Day 9 (**S**) to Day 12–13 (**O**), Day 14–15 (**T**), and Day 16–18 (**F**). All these steps precede the onset of implantation (D19-21).

A characteristic feature of blastocyst development in ruminants (cattle, sheep) and in pig is the elongation process (Figure 1). It is relevant to note that blastocyst elongation is not observed in rodents (mainly mouse, rat) and primates (human, monkey) [5,6]. It is observed in a few ungulates (pig), not in others (horse) however. 

In contrast to cattle, there is an abundance of data on human trophoblast proliferation [7] and differentiation [8] or mouse trophoblast development [9,10]. Cancer research also disclosed an abundance of data on proliferative and invasive properties, thereby providing evidence for molecular circuits shared with human trophoblast cells [7]. Moreover, a special kind of trophoblastic cancer, usually of the placenta - choriocarcinoma type - was used to derive choriocarcinoma cell lines, frequently used to study trophoblast properties (Rcho-1, for example) [11], and to elucidate transcriptional regulation of a bovine trophoblast-specific gene, such as the IFN-tau [12,13]. Stem cell research also provides molecular data on trophoblast stem cells in mouse and human [14,15].

In addition to the text mining issue, we addressed here inter-specific differences in proliferation and differentiation, whose molecular bases are likely common over species and cell types. The total number of published scientific texts on the studied organisms is great; therefore, computer-aided systems must be used for automated knowledge extraction. Our main goal was to search for novel transcriptional regulators involved in the development of bovine extra-embryonic tissues using a new text mining approach (a transcription factor or TF is a protein that binds to specific DNA sequences, activating or inhibiting the recruitment of RNA polymerase to specific genes; see the Transcription Factor Encyclopedia [4]). Text mining in biology is a well-established practice to identify genes and their possible interactants [16,17] that is not much used in animal science [3]. Biological text mining focuses on the following tasks: (1) article classification, (2) protein and gene name recognition and (3) detection of protein-protein interaction pairs [18]. The F-score (balance precision and recall) was around 92% (weighting contribution of many classifiers, from 82 to 87%) for task 2, and the F-score was 29% for task 3 with pattern-based approaches [18]. The event extraction for biological entities became promising for information extraction [19]. Like in a jigsaw puzzle, each document is mined to get a piece of knowledge for a gene identified in sentences in a way to build its context: interactome, localization of gene products, biological processes involving the gene [20-23]. Natural language processing and text-mining of scientific articles can be a tool for digging out a hidden piece of knowledge and for enriching biological data analysis [24]. Our approach is original in the sense that we divide the bulk of literary facts into specialized sub-topics for the topic on which we focused. We introduced the concept of the subcorpus. To do so, at the first step we reduced the list of species to 7 (cattle, human, mouse, rat, sheep, pig and horse) and the list of keywords to 3: trophoblastic (tro), extraembryonic (ex), choriocarcinoma (cho). Our main goal was thus the identification of factors regulating gene expression and growth in the bovine trophoblast as a major contribution to elongation of the blastocyst before its implantation. To achieve this goal, we (i) took into account information from available studies in other species and (ii) identified shared genes or proteins through common Pfam domains (as in [25]) and HomoloGene records. As known, the protein domain is the evolutionary conserved unit of a protein that performs a particular function [26-29] but not all proteins are described by a Pfam.

**Figure 2:**
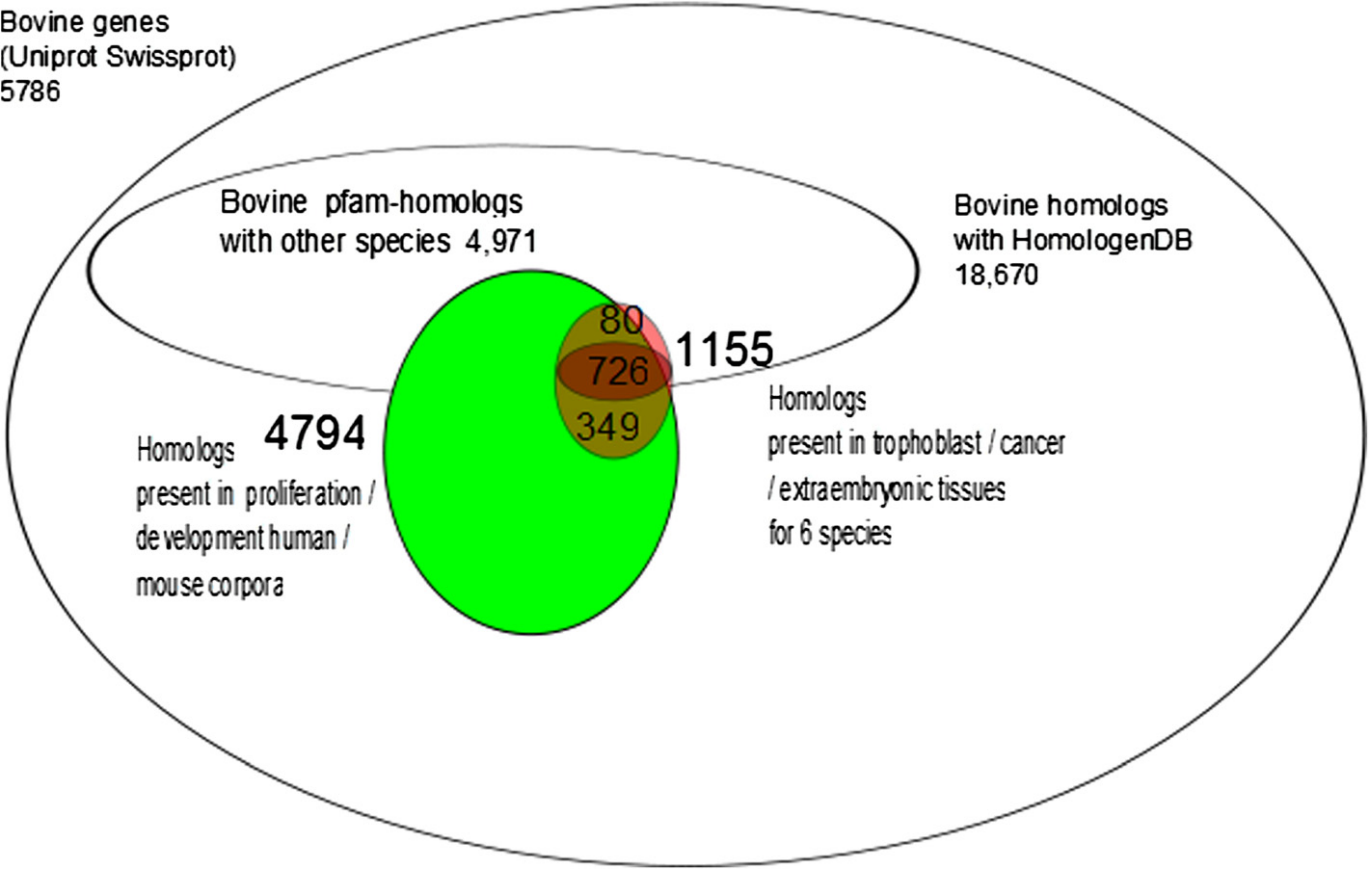
**Hypothesis search space of genes.** Reduction of the hypothesis search space of genes involved in our specific biological issue (symbolised by the red circle). This search space corresponds to 1155 genes: 806 Pfam homologs from the 21 subcorpuses, among which 80 specific to the Pfam database and 726 shared with HomoloGene. The extra group of 349 genes corresponds to the homologs identified by HomoloGene but with no Pfam equivalent within the 21 subcorpuses (details in Tables 1 and 2).

The hypothesis search space was gradually reduced from about 18,670 putative genes (in Homologene/UniProtKB/TrEMBL) to a ranked list of around 1,000 proteins (Figure 2). Ranking was the first step in selecting a pool of genes that can be arranged in an interaction network [26]; the second step was to identify genes, among this top list, involved in regulatory interactions. 

## Methods

### Databases

Our study benefited from concomitance of information scattered over several databases. It is related to a heterogeneous data exploration topic (or *heterogeneous data mining*).

Eight databases were used for processing:

• PubMed and Web of Science

PubMed (formerly Medline) from NCBI is the most scientific text database specialized in biology and medicine; indexing more than 20 million publications in the form of records but recently 30% are available as free full texts. The text database called Web of Science (formerly ISI) from Thomson Reuters consists of more than 45 million publications for any topics, it is mainly used for science assessment. We developed a number of subcorpora about species and biological processes from databases.

• Uniprot

This database from EBI offers knowledge of all the known or putative proteins. Uniprot proposes a file, ‘UniprotKB’, consisting of records for each protein. These were records derived from the TrEMBL database (3,513,283 proteins « not reviewed » hence not completely known) and from the Swiss-Prot database (155,669 proteins « reviewed » or definitely described). From this file, we decided to extract codes about “reviewed” proteins and extracted information from each species. Table 1 shows the distribution of genes described with the Uniprot/Swiss-Prot codes per species.

**Table 1:** Distribution of protein/gene IDs

**Files**	**Proteins (Uniprot)**	**Proteins (Pfam)**	**HomoloGene datasets**
SHEEP	447	409	0
HORSE	280	265	0
PIG	1,374	1,264	0
RAT	7,554	6,670	19,921
HUMAN	20,286	16,478	19,062
MOUSE	16,307	13,944	21,076
BOVINE	5,786	4,971	18,670

Distribution per species of the number of proteins/genes having Uniprot IDs (left) and at least one Pfam domain (center) or an HomoloGene item with the Bovine species (right).

• GO

Gene Ontology from a 20 Institutions consortium is a hierarchy of concepts. It can be useful for getting functional annotation as linguistic concept in such a concept tree to understand the role of protein. We used tags of GO included in Uniprot frames.

• Pfam

The Pfam database (or Protein families) from Sanger Institute consists of at least 12,000 functional domains. A domain is a molecular structure (a DNA sequence associated with a three-dimensional structure), whose properties are preserved over evolution of species and between genomes. Domains contribute to the properties of proteins.

• HomoloGene

HomoloGene is a system from NCBI for automated detection of homologs among annotated genes of several completely sequenced eukaryotic genomes. Together with human, mouse and rat, HomoloGene (Release 66) contains 18,670 bovine genes, placed in 17,472 homology groups.

• GEO

GEO is a database from NCBI of experimental raw datasets, generally microarrays, indexing around 2,800 datasets. We used a dataset named GDS1003 derived from the microarray study of [30] on embryogenesis and early fetal development: time course for bovine embryo [30]. The number of RNA coding sequences was 1,950.

• TcoF-DB

TcoF-DB from King Abdullah University is a database that includes a highly accurate set of 1365 human TFs [31]. Data were extracted from resources: i) a census of human TFs previously published by [32] that was regarded to be a gold standard due to the meticulous way it was created; ii) TRANSFAC, a very well known database on TFs [33]; iii) TFCAT [34] that compiles the mouse TF genes. For these genes, human orthologs were identified by the TcoF-DB team. It must be highlighted that each TF in the TcoF-DB list was curated manually at some point during the data integration.

The crucial step was how to bridge objects from all these independent databases to use information about our starting pool of bovine genes. The object here was the gene (and its product(s), i.e., its protein(s)).

The geneticist usually considers a few differences between a gene and its related protein, which have often the same designation in a publication. We deliberately chose a unique key for a gene as a meta-language between all these databases (gene name, Uniprot ID). This choice was based on (1) usage of both tabular and text databases (2) consensus in the bioinformatics community of researchers, ‘molecular biologists’, that the Uniprot database is universal. For instance, gene names in the microarray data of [30] were transformed into Uniprot IDs using the tool available at http://niaid.abcc.ncifcrf.gov/. In contrast to Uniprot ID, which is unique to a protein, a gene name may not be used regularly in its form; this is why synonyms of gene names must be taken into account (some synonyms are cited in the Uniprot database).

### General analytical workflow for gene identification

Our objective was to identify and classify the proteins involved in trophoblast development. The initial search space consisted of the 18,670 hypothetical bovine proteins (Figure 2). We chose the genes well-described in the Swiss-Prot database, not more than 5,786.

At the next step, we looked through the functional domains of the bovine genes to identify genes from other species that shared the same domains. They were considered as homologs, since they performed probably the same operating functions. In this way, we further reduced the hypothesis search space to about 4,971 for human and mouse. Finally, the literature concerned with genes was taken into consideration in the workflow to infer information for some genes active in embryonic development or proliferation. To do so, we had to build corpora for each species and for each process (i.e., subcorpora).

**Figure 3:**
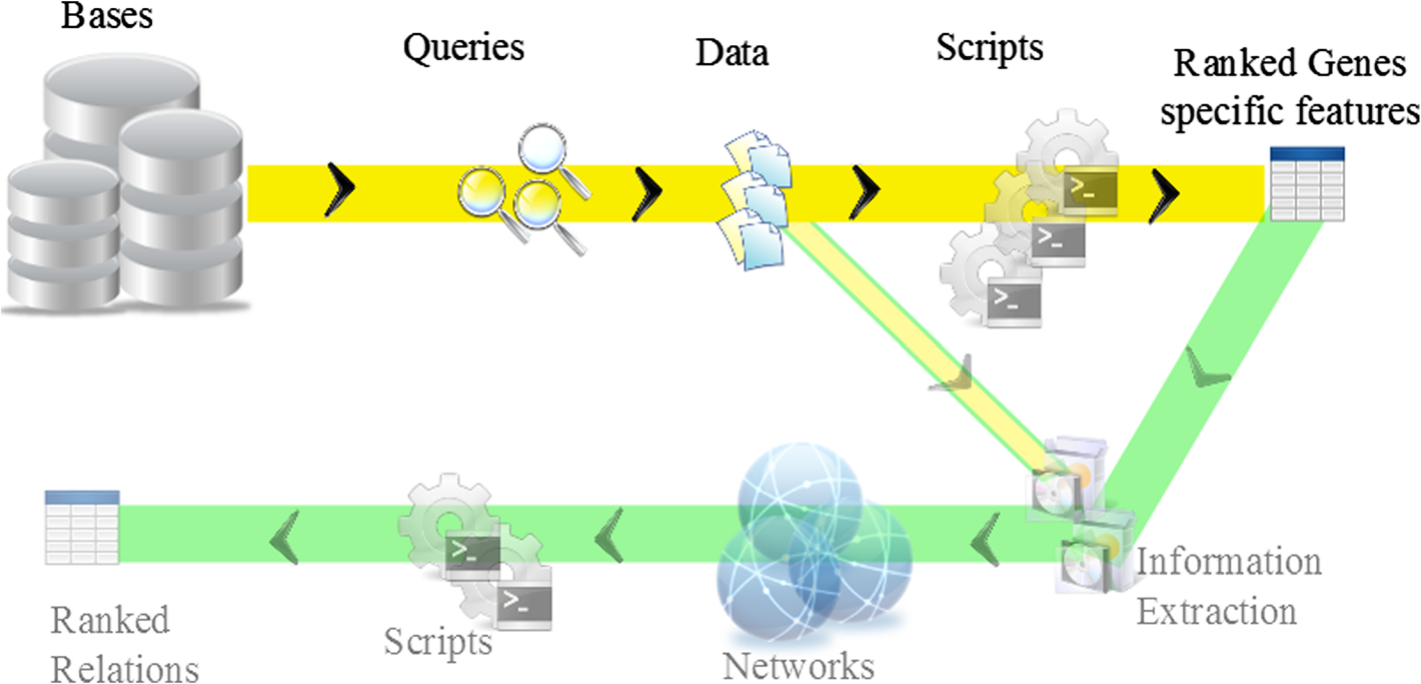
**General workflow.** General workflow separated into two branches.

Figure 3 shows a rough workflow that has two parts. The first branch (yellow) got data from the database to compute the Pfam homologs. They were exported by ranking according to scores. The second branch following the first is beyond the scope of this paper. The list of genes was input to organize their links as an associative network (green branch) taking into account also the same subcorpora used in the yellow branch.

**Figure 4:**
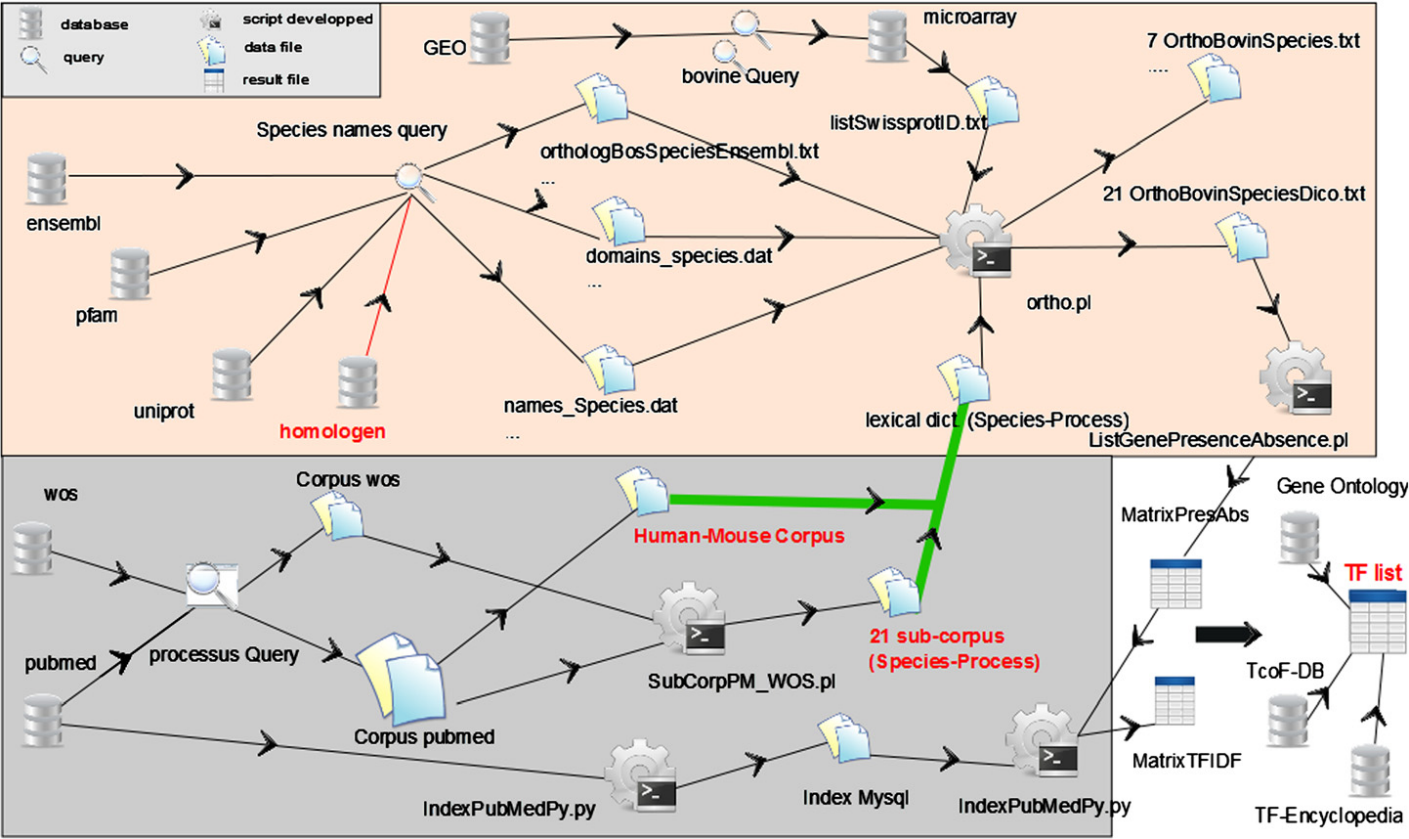
**Detailed workflow.** Workflow: from databases to ranking of relevant genes. Grey: building of sub-corpus. Rose: the Pfam and HomoloGene homologs extraction. The result with transcription factors (TF) list exportation is in white. Green link is the communication between text processing and other databases processing.

Details of the yellow branch are given in Figure 4. In the upper part (rose frame) the workflow has the objective to get data and make feature selection from raw data and encyclopedic data from heterogeneous databases described above. Genes to be analyzed are identified in both Uniprot/Swissprot and high-throughput data concerning the target species, in our case *Bos taurus*. At this step, queries to extract data files were names of species. The script to combine information is called *ortho* and is written in Perl. It generated homologs with the help of functional domains from the Pfam database between cattle and each species, pairwise, in separate exported files. Descriptions were from the collections of publications for which queries were defined by the process names. Ortho script was also able to compute the most representative Pfam IDs of each species (exclusive domains relative to other species and most frequent domains). The matrix of Boolean vectors for the presence or the absence of the 806 Pfam homologs within the 21 corpora was generated.

### Identification of transcription factors among the top genes

a) 11 to 12 TF in the 50 top genes with the Pfam

Transcription factors are proteins that bind to specific DNA sequences, thereby controlling the flow (or transcription) of genetic information from DNA to mRNA. The characteristic feature of TFs is that these proteins possess a sequence specific DNA binding domain. Two approaches were combined to identify TFs among the 50 top genes. First, the gene functions given by GO were analyzed. The presence of GO terms “sequence-specific DNA binding transcription factor activity” “transcription regulator activity”, “bHLH transcription factor binding”, “transcription activator activity”, “DNA binding” was considered as an evidence that the protein is a transcription factor or is involved in some way in transcription regulation. Taking into account the fact that GO annotations are incomplete and in the majority of cases, inferred by electronic annotation, we made also use of the TcoF-DB set of 1,365 human transcription factors which was created very accurately [31]. For these human TFs from the TcoF-DB collection, bovine orthologous genes from each 50 top gene sets were identified. Further manual curation of revealed candidate proteins demonstrated that the results of the two approaches were in a good agreement (Additional file 1).

b) around 150 TF among the 1,155 genes from the TF-IDF-21subcorpora (HomoloGene): we used the TcoF-DB and TF encycopledia to identify the TF families present in this gene sampling, out of the 3 to 400 DNA binding families identified nowadays [4].

c) about 500 TF within the 4,794 genes in the TF-IDF human/mouse corpora (HomoloGene), using the same tools: TcoF-DB and TF encycopledia.

## Results

### Identification of Pfam homologs

To make the information from the model species applicable to cattle, we defined homology between bovine proteins and human, mouse, rat, sheep, pig and horse proteins using Pfam functional domains. These domains are the amino acid sequences, whose structure and role in protein function remained stable during species evolution. Table 1 presents the number of proteins stored in the Pfam database for the 7 species listed above. Clearly, it seemed at first that most proteins of the tabulated species were described by Pfam domains.

The solution suggested for the detection of proteins with functions similar to those of cattle (the Pfam homologs) is based on (1) consideration of the bovine genes whose proteins have at least one Pfam domain and (2) search for proteins of other species, which share all the Pfam domains of a given bovine protein.

**Figure 5:**
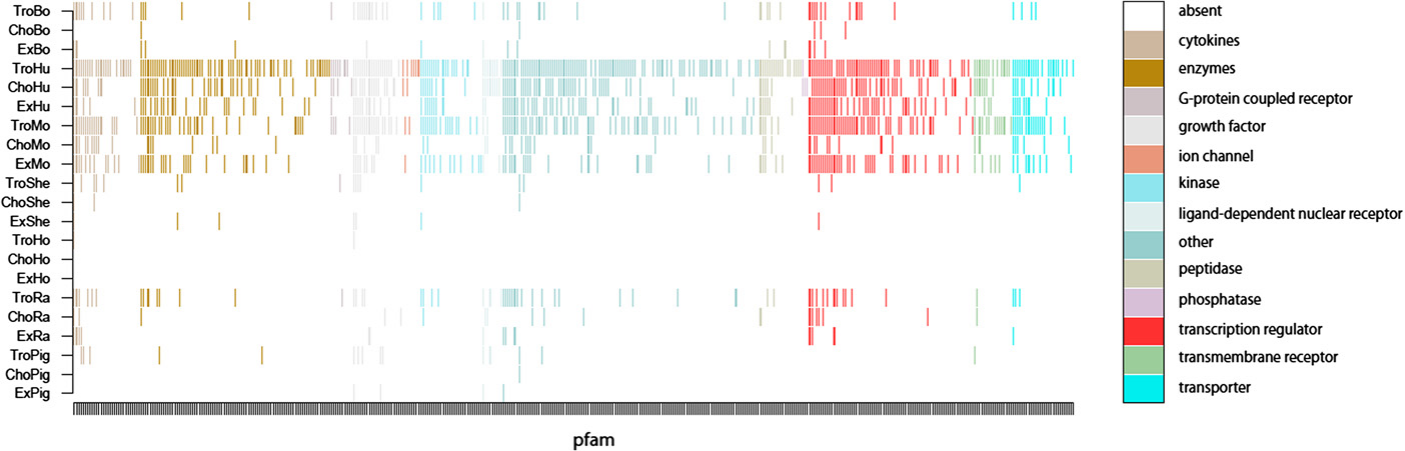
**Distribution of gene categories.** Distribution of gene categories among corpora and species we studied. Gene categories are defined by the IPA tool on the 448 orthologs (among 806) we identified. Transcription regulators are in red. (Display has been realized with R-project [35]).

Figure 5 presents the distribution of the number of *Bos taurus* proteins having Pfam homologs in the examined species. As seen in the figure, from 75 to 85% of the bovine proteins have human, mouse and rat homologs. The number of Pfam homologs was smaller in sheep, horse and pig because the number of protein sequences for sheep, horse and pig was small in Uniprot. It is also seen that virtually all the proteins known in these species had homologs among cattle proteins. Thus, the functional similarities between the proteins with defined sequences from different species appeared quite amply characterized relying on Pfam.

The same Pfam domain could be present in many different proteins in a species. Say, the bovine protein *ACOD_BOVIN* could have the *PF00487* domain. It occurred in 27 different proteins in sheep, rat, pig, human and mouse. Obviously, a group of homologs could contain both orthologs and paralogs. However, we were interested in genes whose products could perform a similar function in different species. Their evolutionary relationships and consequently classification of homologs according to orthologs and paralogs were disregarded. It may be assumed that common Pfam domains justified the reference of proteins to the same functional family and, accordingly, supported their potential involvement in the same biological processes.

### Identification of homologs (HomoloGene)

Homologs from HomoloGene are computed according to gene families. Only Rat, Human and Mouse species describe families in common with the Bovine species. Intersection between families ID is computed to export lists of homologs between Bovine and Human, Bovine and Mouse, and Bovine and Rat. Table 1 shows amounts of homologs between these three species and *Bos taurus*.

### Filtering by a document corpus

Proteins consisting of a single Pfam domain were more numerous than the total number of Pfam IDs describing all the protein domains of a species (for example, for mouse 9,305 against 3,978). Therefore, we had more than one Pfam homolog per protein. It seems plausible that not all the Pfam homologs of a particular protein act in the same tissues of a model organism and participate in similar processes. This demonstrated that the Pfam database alone did not solve the problem of the one-to-one correspondence between bovine and model organism proteins. Thus, additional tools to reduce the number of Pfam homologs were required. Our hypothesis space consisted of seven species (cattle, pig, sheep, horse, human, mouse and rat) and three tissue types (extraembryonic tissues, trophoblast, and choriocarcinoma). Taking this into account, 21 subcorpora were created (see Table 2).

**Table 2:** Number of documents

**Corpus**	**Description**	**PubMed**	**WoS**	**Fusion**
Tropho	Corpus about trophoblast, extraembryonic tissues and choriocarcinoma	20,132	25,702	33,798
HuMo	Corpus about human/proliferation and mouse/development	111,862		
**Subcorpus name**				
TroBo	trophoblast and bovine	409	473	583
ChoBo	choriocarcinoma and bovine	33	27	39
ExBo	extraembryonic tissues and bovine	47	39	58
TroHu	trophoblast and human	4,976	4,762	6,336
ChoHu	choriocarcinoma and human	2,023	1,745	2,553
ExHu	extraembryonic tissues and human	519	417	623
TroMo	trophoblast and mouse	1,724	1,478	2,064
ChoMo	choriocarcinoma and mouse	268	181	308
ExMo	extraembryonic tissues and mouse	1,081	928	1,252
TroRa	trophoblast and rat	564	492	701
ChoRa	choriocarcinoma and rat	156	123	191
ExRa	extraembryonic tissues and rat	124	90	154
TroHo	trophoblast and horse	59	66	82
ChoHo	choriocarcinoma and horse	5	3	6
ExHo	extraembryonic tissues and horse	8	7	12
TroPig	trophoblast and pig	206	178	263
ChoPig	choriocarcinoma and pig	8	4	8
ExPig	extraembryonic tissues and pig	29	28	36
TroShe	trophoblast and sheep	376	357	505
ChoShe	choriocarcinoma and sheep	21	19	25
ExShe	extraembryonic tissues and sheep	44	47	60

Number of processed documents per corpus and subcorpus.

The following query was defined to generate the corpora from the PubMed and the WoS databases:

"((trophoblast* or choriocarcinoma* or extra-embryonic* or extraembryonic*) and (bovine or human or pig or sheep or mouse or rat or horse))"

PubMed is specialized in biomedical documents, but use of WoS is quite complementary and enriches by 20% the initial corpus. The first script was written, considering keywords from the query, to divide these two global corpora (WoS and PubMed) into 21 subcorpora. The second script was used to merge each subcorpus pair (PubMed and WoS) into a single subcorpus avoiding document duplicates. Additional file 2 shows the number of documents per subcorpus and the original global corpus (‘Tropho’). We noticed that some subcorpora were poorly documented including ‘ExBo’, ‘ChoHo’ or ‘ChoPig’ (Additional file 2).

However, as reported by [3], using pig, sheep or horse species in this study likely limited the size of information to be mined. Therefore, a more general corpus has also been created, including only the human and mouse model species. Firstly a query for human has been created to generate a subcorpus called “Human Proliferation” from PubMed. It contained 77,333 documents. Keywords used were:

Query = #req1 OR #req2 OR #req3 OR #req4

#req4 = human AND embryo Field: Title/Abstract, Limits: Humans

#req3 = human AND embryo Field: MeSH Terms, Limits: Humans

#req2 = human AND placenta AND cancer Field: Title/Abstract, Limits: Humans

#req1 = human AND placenta AND cancer Field: MeSH Terms, Limits: Humans

A second subcorpus called “Mouse Development” had also been generated from PubMed. It contained 34,529 documents. Keywords used were:

Query = #req1 OR #req2

#req1 = mouse AND embryo Field: Mesh Terms, Limits: Animals

#req2 = mouse AND embryo Field: Title/Abstract, Limits: Animals

Globally their fusion led to a dictionary of 256,133 tokens.

We then considered the frequency of occurrence, in the publications, of words with similar meanings. For example, extraembryonic is a rare word in the literature concerning cattle since the word “trophoblast” is more often used to designate the extraembryonic tissues. Indeed, the latter word comes from the mouse literature. The word “choriocarcinoma” is not very much applied to livestock species, since it comes from the medical community. Indeed, no choriocarcinoma has ever been described in these species (cattle, sheep, pig, horse), although (i) ectopic grafts of pig trophoblast cells seemed to adopt a tumorigenic phenotype (ii) a rat choriocarcinoma-derived cell line (Rcho-1) as well as (iii) human choriocarcinoma-derived lines (JEG-3, JAR, BEWO; http://www.cell-lines-service.de) were established. As mentioned above with reference to the processing of general corpora, from each subcorpus a dictionary of unique lexical units (i.e., tokens) was established. Additional file 2 shows the number of such units in each subcorpus. Each dictionary was then used to filter (by presence/absence) the list of Pfam homologs.

As known, some genes may have several functions and act in different tissues. When applied to a process, this filtering may be regarded as heuristic, (useful for knowledge discovery), ensuring filtering of genes playing a role in the process: trophoblast development, for example (Table 3) shows the number of Pfam homologs filtered by subcorpora). The whole set of Pfam homologs with the bovine domains were present in all corpora, that is to say, in all tissues and species tested, and was composed of 1,155 genes and 153 TF (Additional file 3). We observed that usage of synonyms improved the search in dictionaries and increased the number of detected genes (Table 3, right column). The list of genes identified in the text was enriched by 30%.

**Table 3:** Number of Pfam homologs per subcorpus

**Subcorpus name**	**Pfam homologs with synonyms**	**Pfam homologs (without synonyms)**
TroBo	**108**	87
ChoBo	**15**	10
ExBo	**35**	35
TroHu	**551**	380
ChoHu	**297**	193
ExHu	**167**	152
TroMo	**336**	241
ChoMo	**74**	51
ExMo	**199**	146
TroRa	**120**	74
ChoRa	**43**	27
ExRa	**28**	16
TroHo	**2**	4
ChoHo	**0**	0
ExHo	**0**	0
TroPig	**24**	18
ChoPig	**2**	1
ExPig	**5**	6
TroShe	**29**	23
ChoShe	**5**	3
ExShe	**8**	9
**Total**	**806**	569

Number of Pfam homologs, with and without Uniprot synonyms.

We envisioned two options to reorganize this list of genes beyond their interaction context. The first relied on identification of their transcription regulatory properties in relation to differentiation pathways. In so doing, we identified 15 to 20% of these homologs as transcription regulators and analyzed their distribution among corpora (Figure 5). Human and mouse corpora were rich in these factors, as compared to the rat or bovine corpora. In contrast, as feared, pig, sheep and horse corpora were poorly documented in this regard. The second option to reorganize the list was related to a sorting operation by score computation. The last one would be a screening for growth factors, cytokines or kinases linked to the cell cycle. We realized that cytokines and growth factors were much less represented than transcription regulators in the main corpora (human, mouse). Interestingly, kinases were similarly well documented in the human and mouse “tro” corpora but less documented in the “cho” corpora. This may indicate their tissue-specificity and open new areas of data-mining.

### Classification of genes - presence score in subcorpora

The classification is intuitive enough, therefore interpretable, and easily tractable. It is based on the identification of genes typical of processes or species. The key is the presence (or absence) of a gene within a subcorpora. Such a classification is the final result of the general workflow (see Figure 3). For this purpose, we created a matrix of scores. There are **n** genes and **m** subcorpora, let **S** be the matrix of scores of the gene set **G** = {g_1_,…,g_n_} by the set of subcorpora **C** = {c_1_,…,c _m_}. **S**_**ij**_ is defined such as **s**_**ij**_ = 1, if a gene **g**_**i**_ is present in the subcorpus **c**_**j**_, otherwise **s**_**ij**_ = 0. Below we show the algorithm:

For each gene i from 1 to n

For each subcorpus j from 1 to m

Compute s[i,j]

End j

End i

*Compute s*_*i0*_ *= Sum of sij for j = 1,m*

*Sort i = 1,n order by s*_*i0*_*(decreasing)*

Our final filtering step led us to **n** = 806 genes using **m** = 21 subcorpora and the first 50 top genes sorted by rank (**s**_**i0**_ > = 9), were potentially selected by biologists for further validations.

### Classification of genes - frequency score in subcorpora and PubMed

The second approach to sort the gene list was the extraction of the more or less specific genes with regard to the process of interest. This meant study of the specificity of a gene described by one or more subcorpora with regard to the whole space of knowledge (WoS and PubMed). Technically, we assigned a smaller weight to a gene occurring very frequently in PubMed. In our workflow, this concerned the processing part in the grey frame (Figure 4).

In computation, we used the classical score in the information retrieval and called tf-idf [36]. This weight takes into account the relative importance of a term (i.e., word or phrase) in a document (it is the ‘term frequency’ or the ‘tf’ part in the score), with a modulation by inverse of the ratio defined by the total number of documents divided by the number of documents containing the term (it is the ‘inverse document frequency’ or the ‘idf’ part of the score). Normalization of the frequency by the size of the documents ensured comparisons of documents of different sizes. In our case, a term was a gene name with its synonyms. The total number of documents in the PubMed and in the subcorpora was used to work out the tf-idf. For a given gene, name normalization is defined by the number of documents of all subcorpora containing this gene name.

To compute the ‘tf’ part of a score, we proceed as follows. Let ***g***_***i***_ be the name of gene ***i***, ***n***_***i***_ be the number of corpus documents containing ***g***_***i***_, ***j*** be the index of the subcorpus containing at least one document with ***g***_***i***_ and ***N***_***j***_ be the number of documents of subcorpus ***j***. Thus,

$$\;tf_{i}=\frac{n_{i}}{\sum_{j}N_{j}}$$

It is the importance of a gene in subcorpora.

The ‘idf’ part was computed as follows. Let **D** be the number of documents in PubMed, and **GF**_***i***_ be the number of documents in PubMed which contain the name of i-th gene, then:

$$\;idf_{i}=\log\frac{D}{GF_{i}}$$

Finally, the score of classification for gene i is **w**_**i**_ **= tf**_**i**_**·idf**_**i**_.

Below we present the algorithm:

For each gene i from 1 to n

Compute GF[i]

End i

For each gene i from 1 to n

For each sub corpus j from 1 to m

compute n[i,j]

End j

Compute SC = Sum |N[j]| from j = 1,m if n[i,j] is not NULL

Compute tf[i] = n[i]/SC

Compute idf[i] = log(D/GF[i])

Compute w[i] = tf[i]idf[i]

End i

Sort i = 1,n order by w[i] (decreasing)

and the results obtained by both classifications (presence/absence and tf-idf) led to a short-list about the 50 top genes over all subcorpora. Each kind of computed score places different genes at the top of the lists, six genes are common: *feta, cata, ntri, soma, sprc, ty3h*. Interpreting general functions given by Gene Ontology in both lists (computed with the two different scores), we observed that they are very close to our initial issue and indicate development and proliferation, however, few TF were identified in these short lists, thus highlighting the risks of the ranking procedures as well as the risk of treating only an “emerging part” of the datasets for further validations.

### Revealing transcription factors among top-ranked genes

Using dictionaries for Human Proliferation and Mouse Development we obtained - out of 18,670 genes isolated from of the bovine genome - (i) 4,794 orthologs with the 2 “Human-Mouse” subcorpora and (ii) 1,155 candidates using the previous 21 subcorpora. Taking advantage of studies that reported gene expression profiles during the elongation of bovine embryos [5,12,30,37-43], we (i) defined a list of transcription regulators (TR, n = 70) among which we kept the transcription factors only (TF, n = 64; Table 4) and - from the terminology of the data mining domain – (ii) used it as a “gold standard” to assess the quality of our approach. Considering the gold standard gene set and the TF lists that were identified through our analyses, we got the following scores: (i) 95.3% recall with the “Human-Mouse” subcorpora (3 non-retrieved genes: *KLF15, PHLDA11, TBX15* due to unrecognized gene ID depending on the species or databases), (ii) 59.4% recall with the 21 subcorpora (26 non-retrieved genes, likely due to restricted gene resources) and (iii) 17.2% recall using the ‘top 100’ genes from the 21 subcorpora, following a ranking by “presence/absence” within these subcorpora (53 non retrieved genes with the workflow). 

**Table 4:** Biological gold standard for transcription factors

				
ASCL2/MASH2	EOMES	HOXB9	KLF9	SIX2
CDX2	ETS1	HOXC4	MSX1	SIX3
c-fos	ETS2	HOXD10	MXI1	SOX13
CITED1	FOSB	HOXD11	MYB	SOX15
CITED2	FOXA2	HOXD13	MYC	SOX17
DLX2	GATA2	JUN	OSTF1	SOX2
DLX3	GATA3	JUND	OTX2	SP1
DLX4	GATA4	KLF10	PAX9	SPARC
DLX5	GATA5	KLF13	PHB	STAT2
DNMT1	GATA6	KLF15	PHLDA1	TBX10
DNMT3A	HAND1	KLF3	PITX2	TBX15
DNMT3B	HNF4A	KLF4	POU5F1	TBX18
ELF2	HOXA4	KLF5	SALL1	TBX5

64 genes generating our biological Gold Standard for Transcription Factors identified so far as differentially expressed in bovine embryos during the elongation process [mainly [30,37,42].

**Figure 6:**
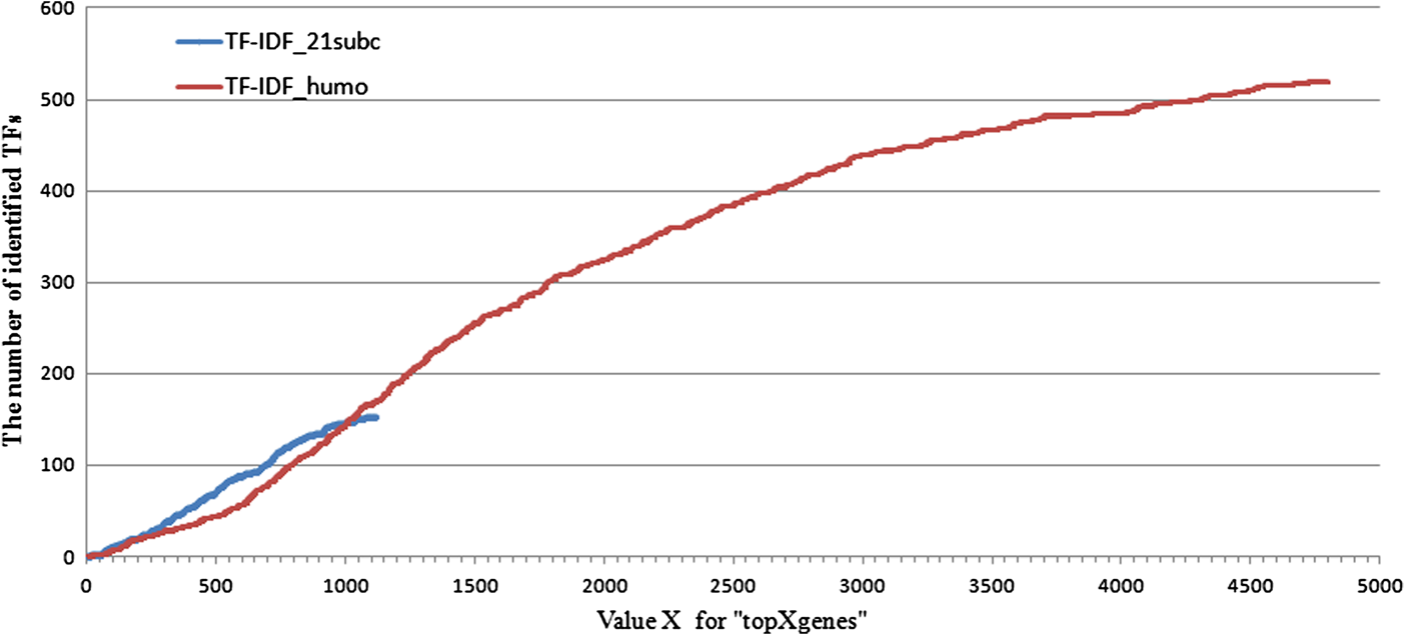
Cumulated curve of TF number in ranked list of homologs.

At first, one could think: “the larger the gene list, the larger the TF content”, however, this is not the case because of a plateau (Figure 6) and 2 TF-identification slopes for the ranges of 0 to 2,500 and 2,500 to 5,000 gene IDs. Moreover, 2 converging arguments came from the literature to reinsure these observations: i) not more than 873 TF were identified over 11,795 genes and 32 human adult tissues, using an Affymetrix gene chip, even if some tissues exhibit more TF than others: brain and placenta for example [4], ii) across 24 eukaryotic genomes from yeast to chimpanzee, not more than 1,391 TF were considered as an evolutionary repertoire of TF [4]. 

**Figure 7:**
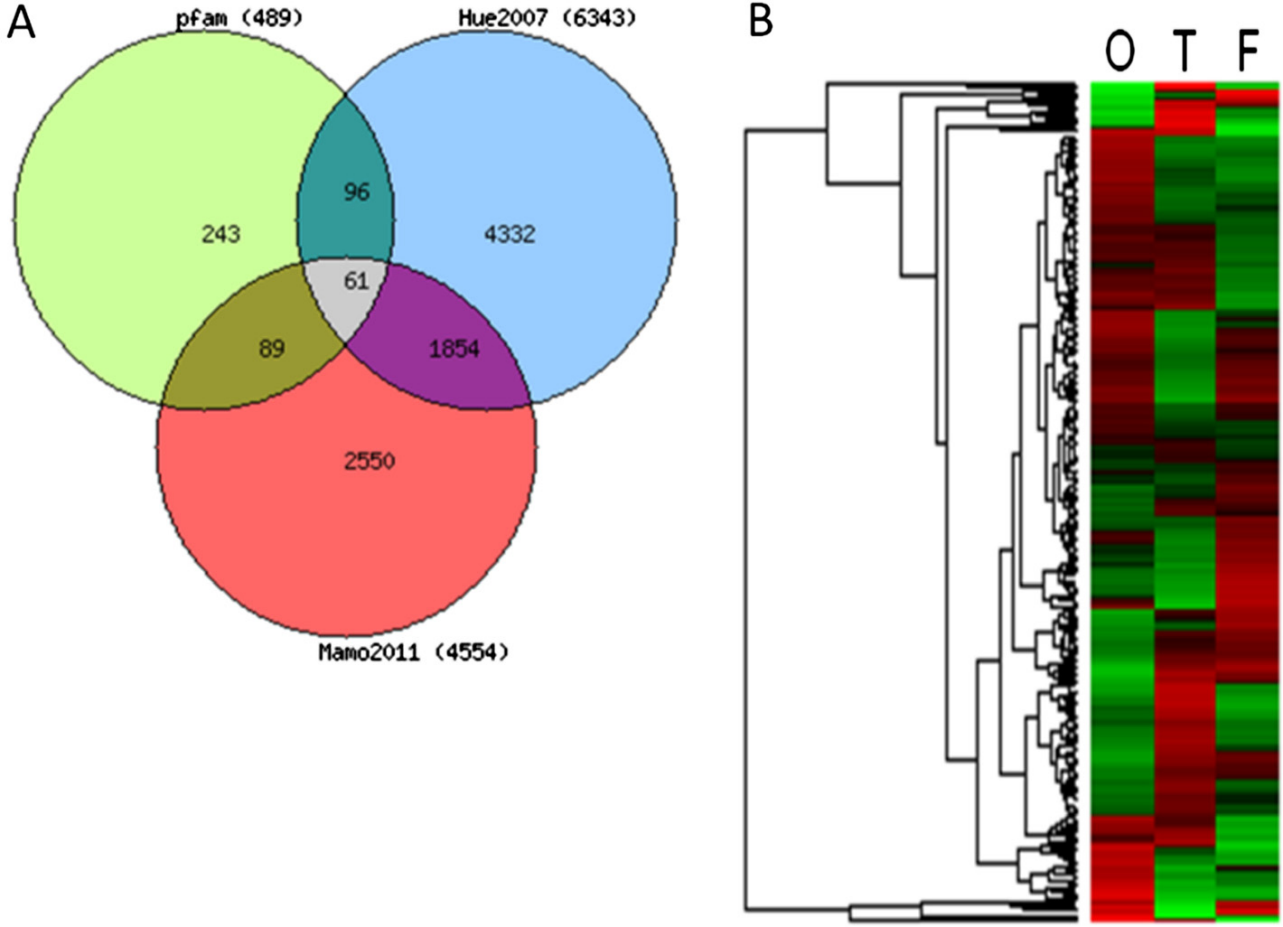
**TF expressed in bovine elongating tissues. A**) Venn diagram including the Pfam-HomoloGene TF from the current work (n=489) from previous studies [41,43]. **B**) Pfam-HomoloGene TF (severine, n=243) as expressed in the array dataset from [41]. O, T and F for: Ovoid, Tubular and Filamentous stages.

**Figure 8:**
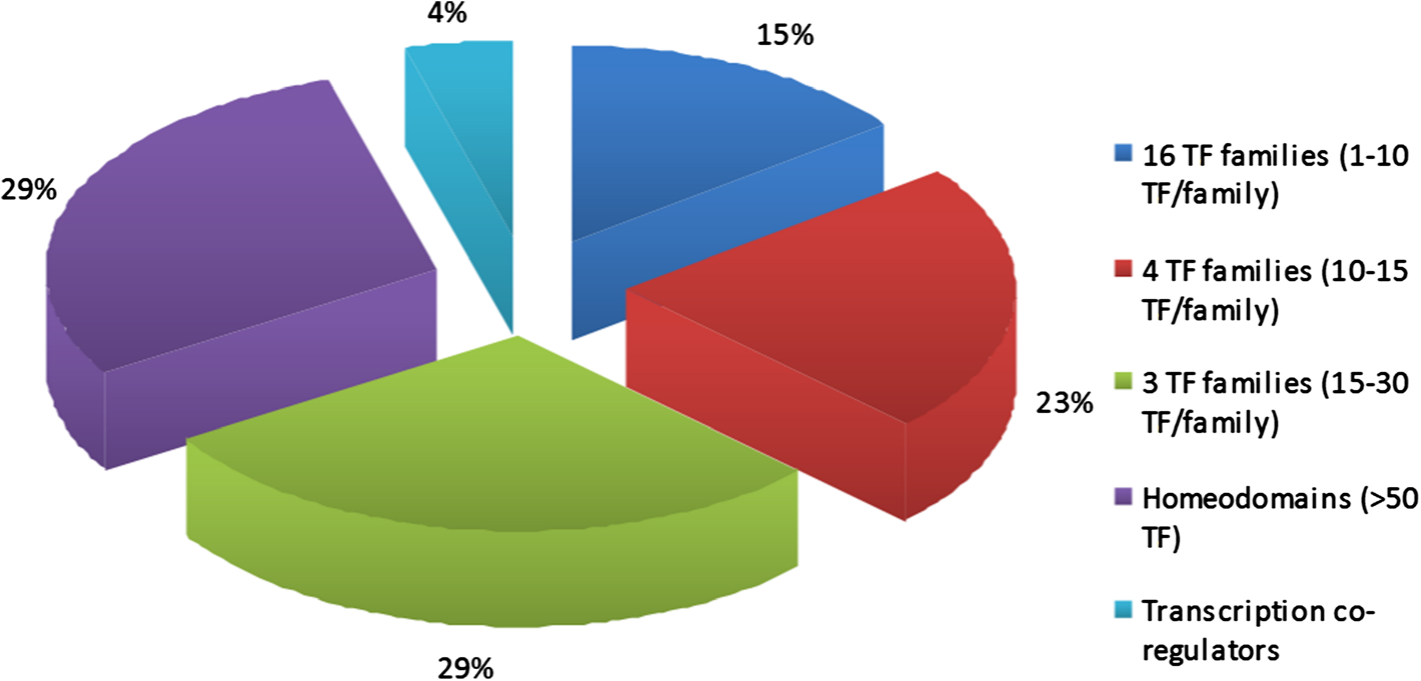
**TF families of 243 putative expressed TF in bovine embryo elongation.** Legend: TF families with 1–10 TF: GATA, GCM, RFX, Stat and T domains, 10–15 TF: HMG, ETS, Leucine zipper, Hormone-nuclear Receptor, 15–30 TF: Forkhead, beta-beta-alpha zinc finger, HLH.

Nevertheless, considering the largest corpus led to the highest recall (95%). Indeed, among the 4,794 homologs, 523 gene IDs were recognized as TF and among these, 489 had a Pfam domain which was used to confirm their main role of “DNA binding” function (Figure 6). Moreover, 246 of these IDs were identified as present within 2 gene expression datasets on bovine elongating embryos (Figure 7). At last, the 243 gene IDs (Additional file 4) that were not in these datasets were however “true” TF since 95% (n = 231; Figure 8) were properly classified by the TF encyclopedia, the other 5% corresponding to unrecognized gene ID or synonyms. Interestingly enough, most of the TF identified here belonged to the homeodomain family (Figure 8), a characteristic family for developmental processes and tissues [4] and a sign of relevant TF classes with our mining approach and workflow. 

However, as underlined by [4], some TF are present in all or most tissues with similar expression levels, thus being ubiquitous TF, while others are selectively expressed in a few tissues, thus bringing specific tissue signatures. Therefore, it is clear that among the 489 TF identified by our approach, only a part will contribute to an extra-embryonic, a trophoblastic or an elongating signature. That is also why the analysis through species and tissues (21 subcorpuses) may help sorting out expression specificities, before further biological investigations. As an example, the transcription regulators from these subcorpuses that did not belong to the “gold standard” revealed interesting biological features: i) **ESR1** is involved in proliferation and development of various tissues [44] and expressed in bovine placenta [45], ii) **HES1** is involved in embryonic patterning [46] and mediates differentiation into mouse trophoblast giant cells [47], iii) **Id2** mediates signaling by activating the proto-oncogene *Myc*[48] and can be down-regulated by TGF-beta signaling to favor differentiation, as evidenced in trophoblast stem cells [49], iv) **NANOG** is required for the maintenance of cellular pluripotency during normal development as well as in cultured embryonic stem cells but has been detected in bovine extra-embryonic tissues [37], v) **P53** regulates the cell cycle and functions as an apoptosis regulator in human villous trophoblast cells [50] and vi) **PHB2** is a transcription coregulator, initially identified as a repressor of estrogen-dependent transcriptional activity. Furthermore, it appeared that all these genes had interesting patterns during elongation with *ID2* increasingly transcribed and *PHB2* decreasingly transcribed from the ovoid to the filamentous stage, whereas *HES1*peaked at the tubular stage.

## Discussion

Elongation, i.e., the lengthening and morphological transition of the conceptus from a sphere to ovoid, tubular and filamentous shapes (Figure 1) provides an increased surface area to enable maternal-conceptus cross-talk and nutrient exchanges [51]. Accompanying elongation is the degradation of the sheath of trophoblast cells covering the embryonic disc (Rauber’s layer) exposing the cells of the embryonic disc to the maternal milieu [52]. The trophoblast is an epithelium and its development combines many biological processes among which proliferation and differentiation. The trophoblast from ruminants does not attach to the uterus of the mother as the trophoblast of rodents and primates do: no invasion, no implantation at a single site and no hemochorial placenta. Total metabolism and protein trafficking are characteristic of the onset of elongation, whereas cellular interactions, cell to cell signaling and cell adhesion become prevalent at the end of it [6,30,53]. Recent results confirmed that an intense multiplication of a non-fully differentiated trophoblast has to be considered at the onset of elongation [37]. However, few data on the molecular bases of this proliferation have been reported in cattle, while they were well documented in such areas as human cancer or human trophoblast development. Since differentiation occurs during elongation and since mouse corpora are well documented for proliferation and differentiation, it was satisfying that our workflow identified on the “human-mouse” corpora the highest number of TF with the best recall of the “gold standard” gene set. Interestingly, the 21 subcorpora identified less transcription factors and genes from the gold standard, but highlighted other genes of interest, such as cytokines or growth factors. Indeed, early implantation is known to be facilitated by an acute inflammatory response of the uterus, a process orchestrated by the trophoblast through the augmentation of cytokine responses [54], and the trophoblast we studied here is only a few days ahead of implantation. The ranking procedures thus helped for gene selection.

Returning to the 806 Pfam homologs, the distribution of genes across subcorpora (Figure 5) reveals that TroHu is close to TroMo, ChoHu and ExMo, suggesting some closeness between species or tissues. Let us assume for further analysis of interactions that (i) species like human and mouse, or bovine and rat can be gathered in only 2 corpora, (ii) tissues may also be gathered or discriminated on the basis of proteins of interest (cytokines or growth factors, for example); All. This could be done to extract common and specific features for sub-classes of species or tissues and refined to screen for:

other genes (cytokines, growth factors, kinases…) or links to other proteins along pathways from the cell membrane to the nucleus

sub-cellular locations through bioinformatics databases [55] or histological atlases [56], hunting for co-expressed genes

and thus, work on text mining data to build interaction maps.

## Conclusions

We created a workflow to search for genes of interest through (1) crossing information from several databases (tables for protein knowledge and raw text) and (2) furnishing a gene list to manage economy in biological testing.

The developed workflow is a mining analytical methodology leading to selection of characteristics of biological processes according to gene and protein properties disseminated in several databases. The originality is highly related to (i) exploration of biomedical text database to make a powerful semantic filter for expression context of genes (ii) use sub-corpora resulting from this filtering and (iii) use fused resources: text and biology. The suggestion to build corpuses, in order not to explore the whole text database, relies on the internal structure of language - based on reuse of words and phrases making them ambiguous; a corpus is a kind of “unstructured” knowledge base of biological facts that may be considered as “cleaned” enough, i.e. containing solely facts about the subject (in our case tissues and species). The ultimate result is a list of Pfam-HomoloGene homologs containing about 15 to 20% of transcription regulators as well as shorter lists of sorted genes (cytokines, kinases…) that could now be the objects of further refined mining.

The workflow can now serve as a new kind of bioinformatics tool to work on fused data sources (raw text and biology) and can thus be useful in studies of a wide range of biological processes.

## Competing interests

The authors declare that they have no competing interests.

## Authors’ contributions

NT, IH, VI, ET designed, performed the study. NT, ET, PD, SAD, EI and DV were involved in data analysis. NT drafted the manuscript. ET, IH, VI, EI and NY corrected the manuscript. All authors read and approved the final manuscript.

## Supplementary Material

Additional file 1 **First ranked genes by tf-idf and presence-absence scores with IPA software comments (location, function).** The colors mark annotation from databases: IPA, red, GO, green. hes1 = hairy/enhancer of split 1, (Drosophila) / ascl2 = Achaete-scute homolog 2 / fos, cfos, AP1 / gata3 = GATA-binding factor 3 / hand1 = Heart - and neural crest derivatives-expressed protein 1 / id2= inhibitor of DNA-binding 2 / Nanog = Homeobox protein NANOG / stat3= Signal transducer and activator of transcription /esr1= / p53=tumor protein / phb2= repressor of estrogen receptor activity (or REA). zinc-finger: GATA3; bHLH: ascl2, hand1, hes1, id2; homeodomain: nanog; ncogene: cFos. Click here for file

Additional file 2 **Dictionaries.** Dictionary sizes (list of all lexical forms) for each corpus and subcorpus. Click here for file

Additional file 3 **Distribution of TF families for 153 TF identified by pfam-homologene and 21 subcorpora.** TF domains were identified with TF encyclopedia. Click here for file

Additional file 4 **List of putative expressed TF for Bovine embryo.** 243 putative TF for bovine elongating tissues; Could however be partly present within the recent RNA sequencing data set, which was published in 2012 and was not included here [57]. In pink, the gene IDs that were not recognized by the TF encyclopedia; in blue, the TF families that were identified through the use of synonyms for these IDs. Click here for file
