# Supplementary figures and images for: Finding biomarkers in non-model species: literature mining of transcription factors involved in bovine embryo development
Source: BioData Min. 2012 Aug 29;5:12. doi: 10.1186/1756-0381-5-12 (PMC3563503; doi:10.1186/1756-0381-5-12)

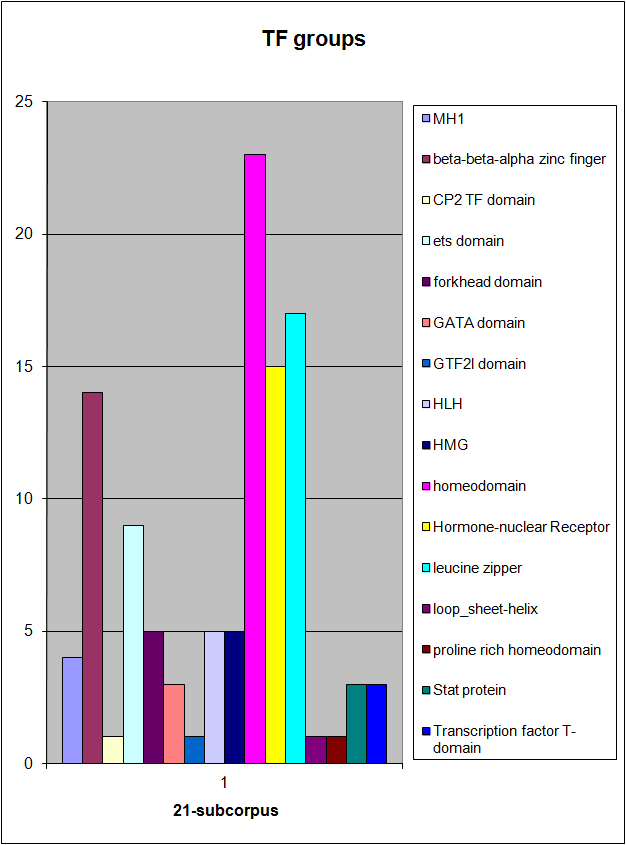

Supplement: Additional file 3 — Distribution of TF families for 153 TF identified by pfam-homologene and 21 subcorpora. TF domains were identified with TF encyclopedia. [file 1756-0381-5-12-S3.png]
